# Understanding the relationship between children's oral health utilization and parent's use of healthcare services

**DOI:** 10.3389/froh.2025.1541045

**Published:** 2025-05-13

**Authors:** Shillpa Naavaal, Rashmi Lamsal

**Affiliations:** Department of Pediatric Dentistry and Dental Public Health and Policy, School of Dentistry, Virginia Commonwealth University, Richmond, VA, United States

**Keywords:** oral health utilization, preventive dental care use, children and adolescents, parent health behavior, Medical Expenditure Panel Survey (MEPS)

## Abstract

**Introduction:**

Parents play an influential role in their children's lives, but little is known about how their healthcare experiences connect. This study examined the relationship between parent's healthcare use and their child's overall and preventive dental care use.

**Methods:**

We pooled three years (2017–2019) of Medical Expenditure Panel Survey (MEPS) data and merged child (0–17 years) and parent data. Our outcomes included any dental visit, preventive visit, and receipt of sealant or fluoride. The primary exposure variable was the parents’ medical and dental care use, grouped into four categories. Descriptive and bivariate analyses were conducted, and multivariable logistic regression analyses were used to examine the associations.

**Results:**

The study included data from 9,927 children. Overall, 50.1%, 42.3%, and 21.2% had any dental visit, preventive visit, and fluoride or sealant application, respectively, in the past 12 months. Among parents, 38.3% had both medical and dental visits, 5.8% had a dental but no medical, 36.7% had a medical but no dental, and 19.1% had neither. Children whose parents had medical and dental visits had more than five times the odds of having any dental visit (aOR = 5.49, 95% CI: 4.64, 6.52) and preventive dental visit (aOR = 5.41, 95% CI: 4.57, 6.39) and 3.64 times the odds of receiving sealant or fluoride application (95% CI: 2.93, 4.53) compared to those whose parents had no dental and medical visits.

**Conclusions:**

Children's oral health utilization is strongly linked with parents’ healthcare use. It can be improved by educating parents and supporting their healthcare use.

## Introduction

Despite advances in oral health care access and delivery in the past decade, one in five 6–11-year-olds and one in two 12–19-year-olds had experienced dental caries, and nearly one in five 6–8-year-olds and 12–19-year-olds (16.4% and 16.6%) had untreated caries, respectively (1). Untreated caries can result in pain, infections, expensive emergency department (ED) visits, and hospitalizations (2) and can negatively impact learning, school attendance, social interactions, and self-esteem (3). The foundation for good oral health starts early in childhood. Still, many children lack routine dental visits, and many do not receive preventive oral health services to support good oral health (4, 5). Acute and unplanned dental care accounts for a loss of 34 million school hours annually among US children (6).

Regular dental care can detect dental issues early and prevent disease progression. The two highly effective evidence-based preventive oral health services to prevent the onset and progression of dental caries among children include fluoride varnish (FV) and dental sealants (7, 8). Sealants are recommended for use on the molars in children and adolescents (9). Dental sealants reduce caries incidence by 80% over two years in the posterior teeth (10). However, despite being a national health objective, sealant receipt is low and disparate (11). During 2011–2016, sealant prevalence in 1st molars and 2nd molars was only 44% and 35%, respectively, among 12–19-year-old children (12), and it varied widely by sociodemographic factors (1).

FV, another proven preventive oral health service, can help remineralize the tooth enamel and protect teeth. It prevents nearly 40% of caries (13). Its use among children is recommended by the American Dental Association, the American Association of Pediatric Dentistry (AAP) (14), and the US Preventive Services Task Force (15). Additionally, the AAP suggests that primary care providers should apply FV on all infants’ and children's teeth at least once every six months, starting when the first tooth erupts and until the establishment of a dental home, but its prevalence remains low (14). A 2014 study using Medical Expenditure Panel Survey data found that only 14.2% of children aged ≤21 years received topical fluoride, sealants, or both (16).

Children and adolescents heavily depend on adults to meet their health needs, including oral health. Parents play a pivotal role in a child's life, and the well-being of children is intricately tied to their parents' physical, social, and emotional health and social circumstances (17). As the primary decision-makers for children, parents' personal choices, experiences, and interactions with health systems can significantly influence and impact the health services used by their children (18). Prior studies have shown an association between parents' healthcare use and children's receipt of well-child visits (19, 20). Evidence also shows a strong relationship between mothers' and children's use of healthcare for physician visits, emergency department use, hospitalizations, vaccination, and mental health visits (21, 22). Similarly, parents who have a negative experience with health care or have problems accessing care may also have difficulty getting care for their children. A recent study using 2016 National Health Interview Survey (NHIS) data found that children had two times the risk of lacking dental visits if the parent had no dental visit and nine times the risk of deferred care if the parent reported inability to afford dental care (23) compared to their counterparts. Similar results were found in another study using older NHIS data (24). However, the relationship between parents' healthcare use and dental care use among children is not known.

Previous studies had used only one measure of dental care use, whether a child had at least one dental visit within the past 12 months, and did not differentiate between dental use and preventive services used, such as sealant and fluoride treatments (23, 24). In this study, we addressed this gap using the Medical Expenditure Panel Survey (MEPS), a preferred survey for estimating health utilization, to significantly extend our understanding of the relationship between parent's medical and dental care use and their child's dental care use. We hypothesized that children whose parent had healthcare use (dental, medical, or both) would have higher odds of dental care use and show a dose-response. For example, dental care among children will be highest among children whose parents had used medical and dental care, followed by those whose parents had just used dental care. Our rationale for these hypotheses is based on the assumption that parents' interaction with the health system in medical or dental settings improves their knowledge about health conditions in general, oral health, and preventive services. By using healthcare services for themselves and understanding the benefits and risks, parents may be more inclined to use these services for their children, influencing children's use of dental care and preventive oral health services.

## Materials and methods

### Data source and study population

We pooled three years (2017–2019) of publicly available MEPS data. MEPS is an annual survey conducted by the Agency for Healthcare Research and Quality, providing a nationally representative sample of the U.S. civilian non-institutionalized population. It includes detailed information about individuals' healthcare use (including visits to a dentist), health insurance status, socioeconomic status, and family characteristics. A single respondent from the household provides information to an interviewer using computer-assisted personal interviewing. More information about MEPS can be found at http://www.meps.ahrq.gov. For a detailed description of the survey and its methodology, see Chowdhury et al. (25). For our study, we merged the household data with the dental visit data each year, and children and parent data were linked to create dyadic observational units.

The unit of analysis for our study was a child. We specifically focused on the youngest child in the family. Our final analytical sample included 9,927 children (weighted *n* = 36,691,423) aged 0–17, with complete information on dental utilization and parent healthcare utilization. We excluded children who resided alone, resided in separate households, or with grandparents (*n* = 1,677), those with non-positive weights (*n* = 368), and older children in the same family (*n* = 10,156).

### Outcome variables

Our analysis included three outcome variables describing the children's dental care use for all children included in the study. Each outcome was categorized as a binary yes/no variable.

Any dental visit: Parents who reported that their child had visited general dentists, dental hygienists, dental technicians, dental surgeons, orthodontists, endodontists, or periodontists in the past 12 months were categorized as “yes” for any dental visit and “no” otherwise.

Preventive dental visit: In the MEPS questionnaire, participants were asked to select all the services they had used during a dental visit in the past 12 months. Parent reported for their child. We constructed the variable to identify children who received preventive dental services if the following procedures were recorded: cleaning, prophylaxis, polishing, periodontal recall, fluoride treatment, or sealant application, and categorized it as “yes” otherwise “no.”

FV/Sealant receipt: This outcome was coded as “yes” if the child received sealant, fluoride, or both. Otherwise, it was coded as “no.”

### Main exposure variable

Parent's healthcare utilization: Our primary exposure variable was constructed using two variables describing medical and dental care use among parents and using the mother as a primary source (21, 22). We used the mother's complete healthcare data when available, and in cases where the mother's data was missing, the father's data was utilized (we used the mother's information for 9,484 children and father's information for 443 children) to construct this variable. A parent was considered to have a medical visit if they had at least one visit to any medical provider, including physicians and non-physicians (e.g., nurses, technicians), in an office-based setting. Similarly, a parent was considered to have a dental visit if they had at least one visit to a general dentist, dental hygienist, dental technician, dental surgeon, orthodontist, endodontist, or periodontist in the past 12 months. Using information from both variables, parent's healthcare utilization was categorized into four groups: had both a medical visit and a dental visit, had a dental visit only, had a medical visit only, and had no medical or dental visits.

### Covariates

We selected the following child, parent, and family covariates based on the prior literature (23, 24): child age (0–5 years, 6–11 years, and 12–17 years), sex (male, female), race and ethnicity [Hispanic, non-Hispanic (NH) White, NH Black, NH Asian, and Other or Multiple races], dental insurance (private dental, public dental, private medical but no dental, and no insurance), number of children under 18 years old in the family (one, two or three, and four or more), and region (Northeast, Midwest, South, and West). The health status of children was assessed three times in a calendar year and was categorized as very good/excellent, good, and fair/poor based on the parent's reports of “excellent or very good” and “good” in at least two rounds, and “fair or poor” otherwise. We did not include the parent insurance variable in the model as it was correlated with the child's insurance variable and was also, in part, accounted for in our main exposure, the parent healthcare use variable.

For children whose mothers' healthcare data was not missing, the mothers' information was used for all parent-level variables. This included variables like parental education, categorized as less than high school, high school, bachelor's (some college or bachelor's), and master's or more; poverty status, categorized as poor (<100% FPL), near-poor (100%–200% FPL), middle income (>200%–400% FPL), and high income (>400% FPL); and employment status, categorized as unemployed, self-employed, or employed. If the mothers' information was missing, the fathers' information was used for these variables.

### Data analysis

We utilized descriptive statistics to provide the demographic, socioeconomic characteristics, dental insurance, and health status associated with children's dental care utilization outcomes. Chi-square tests were used to assess bivariate relationships between outcomes and included variables. As all of our outcome variables were binary, we used multivariable logistic regression models, controlling for various child and parent-related covariates and adjusted odds ratios (aOR), and their corresponding 95% confidence intervals (CI) were calculated. All analyses were weighted and adjusted for the complex survey design using SAS, with survey weights to generate national estimates. Pooled variance structure was used to account for clustering in the panel data. Further details on survey weighting and adjusting for complex design are available on the MEPS website. A *p*-value of 0.05 was considered significant and non-missing data from each variable was utilized for analysis. IRB approval was not necessary as the data is publicly available and deidentified. We also conducted a sensitivity analysis to examine sealant and FV receipt among 6–17-year-olds to align the study population age with the sealant recommendation age (Supplementary Table 1).

## Results

During 2017–2019, 38.3% of US children aged 0–17 years living with a parent, had at least one parent who had both medical and dental visits, 5.8% had only dental visits, 36.7% had only medical visits, and the remaining did not have either visit in the past 12 months (Table 1). Demographically, 40.8% of the children were aged 0–5 years, 51.9% were males, and 50.2% were NH White. Nearly half (48.8%) of children had private dental insurance, 88.1% had a report of excellent/very good health status, and 38.8% resided in the South region. At the family level, 36.7% of children lived in families with income greater than 400% of the federal poverty level, 51.5% had a parent with some college/bachelor's degrees, 69.1% had a employed/self-employed parent, and 56.7% had at least one sibling under 18 years old.

**Table 1:** Characteristics of the study population, Medical Expenditure Panel Survey, 2017–2019 (*n* = 9,927).

Characteristics	Sample size	Weighted % (SE)
Parent healthcare utilization		
Medical and dental	3,240	38.4 (0.75)
Dental and no medical	573	5.8 (0.30)
Medical and no dental	3,834	36.7 (0.70)
No medical and dental visit	2,280	19.1 (0.56)
Age group		
0–5 years	3,953	40.8 (0.80)
6–11 years	3,238	30.5 (0.74)
12–17 years	2,736	28.7 (0.73)
Sex		
Male	5,146	51.9 (0.75)
Female	4,781	48.1 (0.75)
Race/ethnicity		
Hispanic	3,256	24.3 (1.08)
Non-Hispanic White	3,945	50.2 (1.08)
Non-Hispanic Black	1,530	13.5 (0.64)
Non-Hispanic Asian	578	5.9 (0.43)
Other or multiple races	618	6.1 (0.34)
Dental insurance		
Private dental	4,012	48.8 (0.88)
Public dental	4,300	33.1 (0.95)
Private medical but no dental	1,358	15.8 (0.67)
No insurance	257	2.3 (0.21)
Health status		
Very good/excellent	8,445	88.1 (0.46)
Good	1,320	10.6 (0.44)
Fair/poor	159	1.3 (0.15)
Region		
Northeast	1,459	17.0 (1.20)
Midwest	2,032	20.3 (1.08)
South	3,922	38.8 (1.46)
West	2,514	23.9 (1.34)
Family poverty status		
Poor	2,108	12.9 (0.51)
Near poor	2,234	19.5 (0.60)
Middle income	2,869	30.9 (0.66)
High income	2,716	36.7 (0.96)
Parent education		
<High school	1,547	10.4 (0.54)
High school graduate	2,613	20.7 (0.62)
Bachelor's degree	4,398	51.6 (0.80)
Postgraduate degree	1,312	17.3 (0.88)
Parent employment		
Self-employed	779	8.1 (0.42)
Employed	5,565	61.0 (0.77)
Unemployed	3,528	30.9 (0.78)
Parent dental insurance		
Private dental	4,515	53.4 (0.88)
Public*	2,504	19.3 (0.76)
Private medical but no dental	1,746	18.9 (0.68)
No insurance	1,162	8.3 (0.45)
Number of children under 18 years in the family		
One	4,010	43.3 (0.74)
Two or three	5,180	51.0 (0.74)
Four or more	737	5.7 (0.31)

* Public insurance for parents may or may not cover dental care, as Medicaid dental coverage for adults is not the same across all states.

### Bivariate analysis

#### Any dental visits

**Figure 1:**
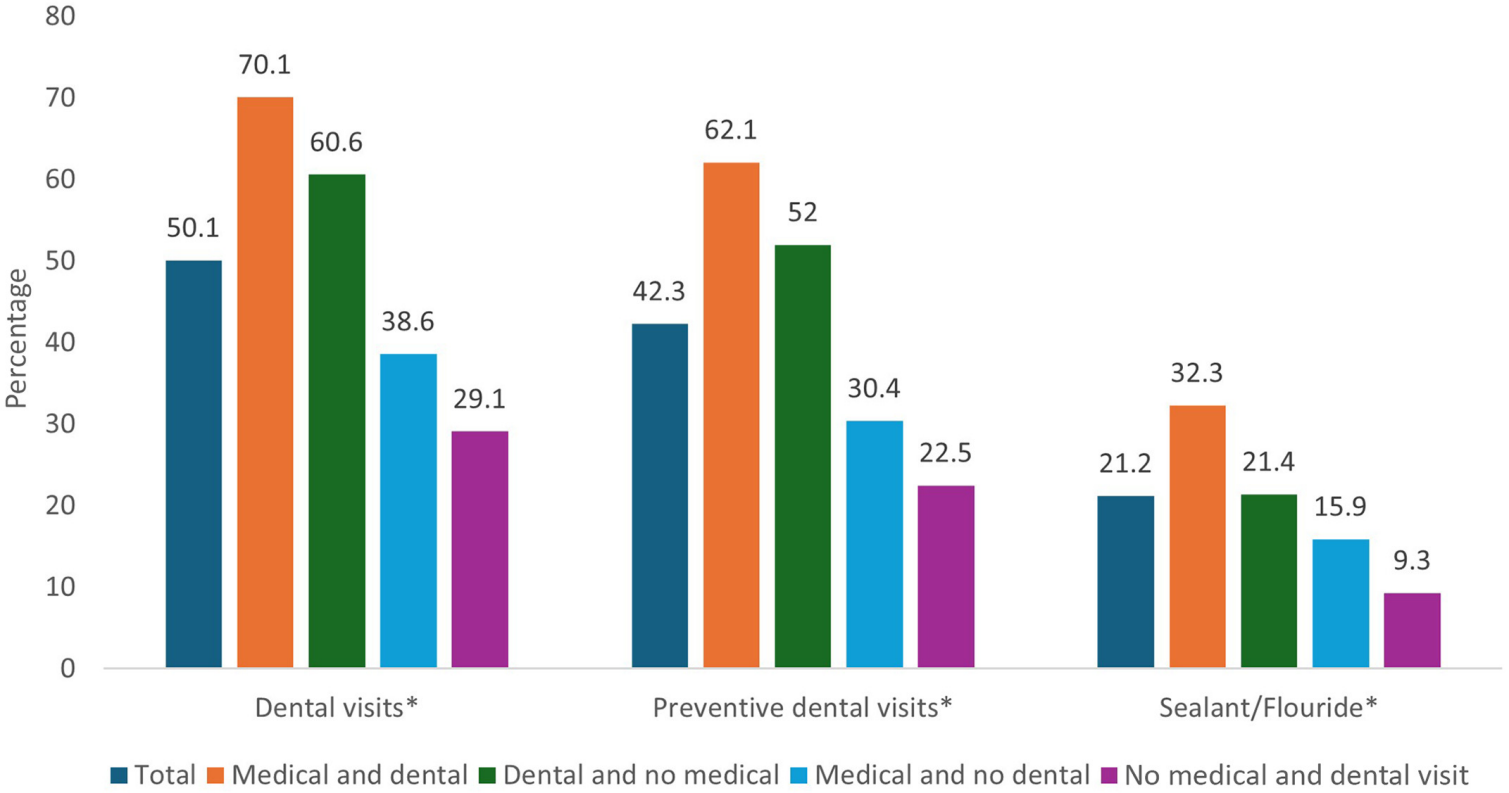
Dental care utilization outcomes among children stratified by parent's health care utilization, Medical Expenditure Panel Survey, 2017–2019. *Statistical significance at *p*-value <0.001.

Overall, 50.1% of children had a dental visit in the past 12 months. Among children whose parents had both medical and dental visits, 70.1% had a dental visit. The percentage of dental visits reduced to 60.6% among children whose parents had a dental visit only, 38.6.% among those whose parents had a medical visit only, and 29.1% among children whose parents had neither medical nor dental visits (Figure 1).

The percentage of dental visits was over 50% among children in age categories 6–11 (60.8%) or 12–17 (61.9%) years, as well as among NH White (54.5%), NH Asian (50.1%), or other races (50.5%) compared to their counterparts (Table 2). There was a difference of 27.0 percentage points in any dental visit between children with private dental insurance (56.9%) and those without any insurance (29.9%). More than 50% of children residing in the West (53.6%), Midwest (50.6%), or Northeast (50.4%) regions had a dental visit (Table 2). The dental visit percentage was higher among children from families with income greater than 400% of the federal poverty level (60.7%), whose parents had a postgraduate degree or more (62.5%), or those who lived in families with 2–3 children (50.6%) compared to their counterparts.

**Table 2:** Bivariate associations between children's dental utilization outcomes and included children, family, and parent characteristics, Medical Expenditure Panel Survey, 2017–2019.

Characteristics	Any dental visits		Preventive dental visits		Fluoride/Sealant receipt	
	Yes	*p*-value	Yes	*p*-value	Yes	*p*-value
Age group		<0.001		<0.001		<0.001
0–5 years	34.0 (1.11)		27.2 (1.02)		14.7 (0.75)	
6–11 years	60.8 (1.19)		54.6 (1.24)		29.0 (1.18)	
12–17 years	61.9 (1.21)		50.7 (1.30)		22.3 (1.23)	
Sex		0.486		0.977		0.883
Male	49.6 (0.99)		42.3 (1.06)		21.2 (0.86)	
Female	50.7 (1.05)		42.3 (1.03)		21.3 (0.90)	
Race/ethnicity		<0.001		<0.001		<0.001
Hispanic	44.6 (1.21)		37.5 (1.20)		17.0 (0.97)	
Non-Hispanic White	54.5 (1.06)		47.1 (1.10)		25.6 (1.02)	
Non-Hispanic Black	43.9 (1.61)		34.3 (1.60)		13.9 (1.27)	
Non-Hispanic Asian	50.1 (2.61)		41.2 (2.72)		15.2 (2.01)	
Other or multiple races	50.5 (2.75)		40.1 (2.65)		24.5 (2.42)	
Dental insurance		<0.001		<0.001		<0.001
Private dental	56.9 (1.00)		48.4 (1.08)		24.9 (0.93)	
Public dental	42.2 (0.98)		34.6 (0.95)		16.2 (0.82)	
Private medical, no dental	48.9 (1.99)		41.8 (1.94)		22.1 (1.63)	
No insurance	29.9 (3.50)		24.3 (3.55)		10.4 (2.50)	
Health status		0.370		0.962		0.777
Very good/excellent	50.5 (0.76)		42.3 (0.82)		21.4 (0.73)	
Good	48.2 (1.67)		41.8 (1.71)		20.1 (1.37)	
Fair/poor	46.1 (5.45)		41.9 (5.30)		21.3 (5.06)	
Region		0.010		0.063		<0.001
Northeast	50.4 (1.76)		42.5 (1.62)		20.4 (1.50)	
Midwest	50.6 (1.27)		44.5 (1.40)		24.9 (1.40)	
South	47.7 (1.27)		40.0 (1.26)		18.3 (1.01)	
West	53.6 (1.29)		44.0 (1.58)		23.5 (1.62)	
Family poverty status		<0.001		<0.001		<0.001
Poor	37.5 (1.30)		29.9 (1.32)		12.3 (0.99)	
Near poor	43.2 (1.29)		35.4 (1.27)		17.5 (1.10)	
Middle income	47.3 (1.13)		40.0 (1.11)		20.2 (0.95)	
High income	60.7 (1.31)		52.2 (1.36)		27.3 (1.20)	
Parent education		<0.001		<0.001		<0.001
< High school	38.8 (1.62)		34.2 (1.57)		13.2 (1.45)	
High school graduate	40.5 (1.32)		33.7 (1.32)		15.0 (1.00)	
Bachelor's degree	52.4 (1.01)		44.1 (1.01)		23.1 (0.89)	
Postgraduate degree	62.5 (1.67)		52.9 (1.69)		28.5 (1.75)	
Parent employment		<0.001		<0.001		<0.001
Self-employed	53.2 (2.34)		45.6 (2.46)		22.8 (2.26)	
Employed	53.4 (0.93)		44.8 (0.99)		23.0 (0.88)	
Unemployed	43.0 (1.17)		36.5 (1.96)		17.4 (0.89)	
Number of children under 18 years in the family		0.034		0.002		0.004
One	50.5 (1.09)		40.9 (1.12)		19.5 (0.99)	
Two or three	50.6 (0.97)		44.1 (0.97)		23.0 (0.86)	
Four or more	43.3 (2.41)		36.0 (2.06)		18.9 (1.90)	

Note: Row percentages are presented in this table. All percentages were weighted, and *p*-values were derived using Pearson's Chi-square statistics. The estimates presented are weighted percentages with standard errors in parentheses.

#### Preventive dental visits

Among this study population, 42.3% of children had preventive dental visits in the past 12 months. When stratified, this outcome followed a similar pattern as “any dental visit” outcome. Preventive dental visits were highest among children whose parents had both medical and dental visits (62.1%) and lowest among those whose parents had no medical or dental visits (22.5%) (Figure 1).

Preventive dental visits were higher among children aged 6–11 (54.6%), NH White (47.1%), those from high-income families (52.2%), or who had a higher educated parent (52.9%) or employed parent (44.80%) compared to their counterparts (Table 2). Children with private dental insurance (48.4%), and those living in families with 2–3 children (44.1%) had a higher prevalence of preventive dental visits than others.

#### FV/sealant receipt

Only 21.2% had FV/sealant application in the past 12 months (Figure 1). FV/Sealant receipt was 32.3%, 21.4%, 15.9%, and 9.3% among children whose parents had both medical and dental visits, had only dental visits, had only medical visits, and had no medical or dental visits, respectively (Figure 1).

Among 6–17-year-olds, the percentage of FV/sealant receipt had a similar pattern: 38.8%, 27.0%, 18.9%, and 10.1% among children whose parents had both medical and dental visits, had dental visits only, had medical visits only, and had neither medical or dental visit, respectively (data not shown).

Among all children, the receipt of FV/sealant was lowest among 0–5-year children (14.7%), NH Black (13.9%), those living in poor income households (12.3%), those with unemployed parent (17.4%) or those whose parent had less than high school educated parents (13.2%). Children who had public insurance (16.2%) or children who had no insurance (10.4%), those who lived in households with four or more kids (18.9%), and those who lived in the South region (18.3%) had a lower percentage of FV/sealant receipt than their counterparts.

### Multivariable logistic regression analysis

#### Any dental visits

Children's dental care use was strongly associated with their parent's medical and dental care use. Children whose parent had both medical and dental visits, had only dental visit or had only medical visit had 5.49 (95% CI: 4.64, 6.52), 3.77 (95% CI: 2.96, 4.78), and 1.51 (95% CI: 1.29, 1.77) times odds, respectively, of having dental visit than children whose parent had no medical and dental visit after controlling for all covariates (Table 3).

**Table 3:** Adjusted logistic regression model examining the association between parents’ healthcare utilization and children's dental services utilization outcomes, Medical Expenditure Panel Survey, 2017–2019.

Characteristics	Any dental visits	Preventive dental visits	Fluoride/Sealant receipt
	OR (95% CI)	OR (95% CI)	OR (95% CI)
Parent healthcare utilization			
Medical and dental	5.49 (4.64, 6.52)***	5.41 (4.57, 6.39)***	3.64 (2.93, 4.53)***
Dental and no medical	3.77 (2.96, 4.78)***	3.64 (2.85, 4.64)***	2.30 (1.70, 3.11)**
Medical and no dental	1.51 (1.29, 1.77)***	1.45 (1.24, 1.71)***	1.66 (1.35, 2.03)***
No medical and dental visit	Ref	Ref	Ref
Age group			
0–5 years	0.28 (0.24, 0.33)***	0.27 (0.23, 0.31)***	0.40 (0.34, 0.47)***
6–11 years	Ref	Ref	Ref
12–17 years	1.19 (1.02, 1.38)*	0.95 (0.82, 1.10)	0.74 (0.62, 0.88)***
Sex			
Male	Ref	Ref	Ref
Female	1.06 (0.93, 1.21)	1.01 (0.889 1.14)	1.00 (0.87, 1.15)
Race/ethnicity			
Non-Hispanic White	Ref	Ref	Ref
Hispanic	1.01 (0.87, 1.17)	0.98 (0.84, 1.15)	0.84 (0.70, 1.00)
Non-Hispanic Black only	0.86 (0.72, 1.02)	0.76 (0.64, 0.90)**	0.62 (0.48, 0.79)***
Non-Hispanic Asian only	0.89 (0.70, 1.13)	0.85 (0.67, 1.08)	0.54 (0.39, 0.74)***
Other or multiple race	1.07 (0.86, 1.34)	0.92 (0.74, 1.16)	1.13 (0.86, 1.48)
Dental insurance			
Private dental	Ref	Ref	Ref
Public	1.15 (0.98, 1.36)	1.15 (0.97, 1.37)	1.17 (0.96, 1.43)
Private health but no dental	0.76 (0.63, 0.92)**	0.81 (0.67, 0.97)*	0.92 (0.76, 1.12)
Uninsured	0.41 (0.29, 0.59)***	0.42 (0.29, 0.63)***	0.50 (0.29, 0.88)*
Health status			
Fair/poor	0.84 (0.50, 1.41)	1.01 (0.61, 1.67)	1.14 (0.61, 2.13)
Good	1.04 (0.89, 1.23)	1.14 (0.96, 1.34)	1.13 (0.94, 1.37)
Very good/excellent	Ref	Ref	Ref
Region			
Northeast	0.85 (0.71, 1.01)	0.85 (0.72, 1.00)	0.93 (0.75, 1.16)
Midwest	0.94 (0.81, 1.09)	0.99 (0.85, 1.15)	1.22 (1.00, 1.48)
South	Ref	Ref	Ref
West	1.17 (1.01, 1.36)*	1.03 (0.87, 1.23)	1.26 (0.99, 1.60)
Family poverty status			
Poor	0.83 (0.67, 1.04)	0.73 (0.58, 0.91)**	0.67 (0.50, 0.91)*
Near poor	0.85 (0.69, 1.05)	0.80 (0.65, 0.98)*	0.88 (0.70, 1.11)
Middle income	0.80 (0.68, 0.94)**	0.81 (0.69, 0.95)**	0.86 (0.73, 1.02)
High income	Ref	Ref	Ref
Parent education			
<High school	0.57 (0.45, 0.72)***	0.79 (0.62, 1.00)	0.61 (0.43, 0.88)**
High school	0.63 (0.52, 0.76)***	0.75 (0.61, 0.91)**	0.67 (0.52, 0.86)**
Bachelor's degree	0.77 (0.65, 0.92)**	0.85 (0.72, 1.00)	0.86 (0.70, 1.05)
Postgraduate degree	Ref	Ref	Ref
Parent employment			
Unemployed	0.95 (0.82, 1.09)	1,01 (0.88, 1.16)	0.96 (0.81,1.13)
Self-employed	1.02 (0.83, 1.26)	1.07 (0.86, 1.33)	0.99 (0.76, 1.28)
Employed	Ref	Ref	Ref
Number of children under 18 years in the family			
One	Ref	Ref	Ref
Two or three	1.32 (1.16, 1.51)***	1.46 (1.28, 1.67)***	1.34 (1.12, 1.58)***
Four or more	1.63 (1.29, 2.05)***	1.62 (1.30, 2.02)***	1.52 (1.13, 2.03)**

Statistically significant *p* values are indicated as *p < 0.05, **p < 0.01, ***p < 0.001.

In the adjusted model, we also found that younger children, those without dental insurance, those who had parents with a bachelor's or less education, were less likely to have a dental visit compared to their respective counterparts. Children residing in the West region and those living in households with one or more siblings had higher odds of dental visits than children living in the South region and those living in single-child households, respectively.

#### Preventive dental visits

Children whose parents had medical and dental visits had 5.41 times odds of having a preventive dental visit (95% CI: 4.57, 6.39) than children whose parents had no healthcare visits. Similarly, children whose parents had only a dental visit had 3.64 times the odds (95% CI: 2.85, 4.64), and those whose parents had only a medical visit had 1.45 times the odds (95% CI: 1.24, 1.71) of a preventive dental visit compared to children whose parents had no medical or dental visits (Table 3).

Results in the preventive visit model were similar to “any dental visit” model for other included covariates, with a few exceptions. NH Black children had 24% lower odds (aOR = 0.76, 95% CI 0.64, 0.90) of preventive dental visits than NH White children. Children living in middle-income, near poor or poor had lower odds of preventive visits than those living in high-income. Region, adolescent age group (12–17 years), less than high school and bachelor's degree were not found to be significantly associated with the preventive dental care use in the model.

#### FV/sealant receipt

The multivariable logistic regression model in Table 3 for sealant/FV receipt showed that children whose parents had both medical and dental visits, had only dental visits, or had only medical visits had 3.64 (95% CI: 2.93, 4.53), 2.30 (95% CI: 1.70, 3.11), and 1.66 (95% CI: 1.35, 2.03) times odds of FV/sealant receipt compared to children whose parents had no medical or dental visit (Table 3). Receipt of FV/sealant was lower among 0–5- and 12–17-year-old children compared to 6–11-year-olds. NH Black, NH Asian children, those who had parents with high school or less education, those who lived in poor-income households, and those who were uninsured had lower odds of FV/sealant receipt. Children living in families with two or more children had higher odds of FV/sealant receipt.

The sensitivity analysis for sealant/FV receipt among 6–17-year-olds showed results in a similar direction and stronger magnitude than among 0–17-year-olds for all variables (Supplementary Appendix Table 1).

## Discussion

During 2017–2019, among 0–17-year-old children, one in two (50.1%) had a dental visit, 42.3% had a preventive dental visit, and only 21.2% received an FV/sealant application. Nearly 85% of children who had any dental visit had received preventive services (42.3%). Still, only half of those who received preventive services received FV/sealant (21.2%), which varied by parent healthcare use. Dental visit, preventive dental visit, and sealant/FV application among children whose parent had both Medical or dental visits were 9.5–10.9 percentage points, 16.4–31.7 percentage points, and 23.0–41.0percentage points higher compared to children whose parents only had a dental visit, had only a medical visit or had no medical or dental visit, respectively in the unadjusted data. Adjusted regression models showed similar results with nearly 3–5 times the odds of any dental visit, preventive visit, and sealant/FV receipt among children whose parents had both medical and dental visits, 1.8–2.8 times the odds among those whose parents had dental visit only and 1.3–1.5 times the odds among those whose parents had medical visit only compared to children whose parents had no dental or medical visits. The data finding supports our dose-response hypothesis for all outcomes.

Among our study population, 19% of parents did not have dental or medical visits in the past 12 months, and more than 1/3rd had a medical visit but not a dental visit. Given the positive association between parent's and child's dental visits and that more than 50% of parents did not have a dental visit in the past 12 months, it is necessary to improve parent's utilization of dental care, not only to support their oral health but also to improve dental care use among their children, especially for preventive care and evidence-based dental services. Sealants and fluoride varnish are proven effective in arresting and preventing caries (7, 13) and reducing the burden of dental caries in the long term. The improvement in preventive care and FV/sealant use among children whose parents had dental care use was nearly twice or more compared to children whose parents had no dental and medical care use, showing evidence of parents' role in influencing their child's preventive dental care use.

One way to support dental care utilization among adults is to provide coverage through Medicaid. Many adults lack dental insurance, and cost remains a prime barrier to dental care access and utilization (26). Several states have expanded dental coverage in the past few years, but many still do not cover dental services beyond emergency services (27). A study found a 5-percentage-point reduction in the prevalence of untreated caries among children after Medicaid-enrolled adults had access to dental coverage for at least one year (28).

We found that parents' medical care use alone was also strongly associated with children's dental care use. One explanation for this could be the increased awareness and knowledge among parents who seek health care. These parents may be better able to navigate health systems and prioritize their children's health, including oral health, due to their exposure to the health system. To further support parents in investing in their child's oral health, discussing oral health at regular adult medical visits could be valuable. Primary care providers can conduct oral health screening, provide fluoride varnish, and refer young children to a dentist (29), but this is not a practice during adult visits. Furthermore, it's important to note that parents are role models for children and can inculcate healthy behaviors and knowledge in the family unit, and thus, children may adopt similar behaviors as they grow.

Additional findings show that receipt of FV/sealants was lower among 0–5-year-olds and 12–17-year-olds compared to 6–11-year-olds. In the 0–5 age group, FV mainly drives the results as these children do not have permanent molars that are eligible for sealants. The sensitivity analysis showed that 12–17-year-olds, a group eligible for both FV and sealants, also had lower odds of FV/sealant receipt compared to 6–11-year-olds. Molars are the most susceptible teeth for dental caries (30). Sealants are typically recommended for permanent first and 2nd molars (6 years of age and above), and fluoride can be applied since the first tooth erupts (6 months of age and above). Our findings highlight the need to target and improve evidence-based oral health services delivery to both younger and older age groups while maintaining the oral health of 6–11-year-olds. School oral health programs are effective community oral health programs that can deliver FV and sealants to elementary school-age children who otherwise would not receive them. Still, these programs are not available to all and are uncommon among higher grades and in the preschool/Head Start population. Expanding these programs and educating parents and children about the benefits of preventive oral health services could help to improve oral health among these age groups. Additionally, increasing the delivery of FV among young children by primary care providers could be another way to increase the utilization of effective oral health services.

In our study, we found that the FV/Sealant receipt was lower among NH Asian and NH Black children compared to NH White children, uninsured children, and children whose parents had high school or less education compared to their counterparts. These findings concur with the published literature (24). However, our study found that children with public insurance were not significantly different from private insured children in having any dental visit, preventive dental visit and FV/Sealant receipt in the adjusted analysis. This is an encouraging finding suggesting improved oral health and health equity among children with public insurance. One of the potential reasons for these findings may be the use of sealant and dental care use measures as national health objectives and the endorsement of oral evaluation, topical fluoride, and sealant receipt in the CMS child core set measures and by the National Quality Forum (11, 31).

We also found that families with more than one child used any dental care, preventive dental care, and received FV/sealant more frequently than those with only one child. One explanation for this finding may be the older child's oral health experience and familiarity with the health system, which may encourage timely and preventive oral health use and maintenance of good oral health.

To our knowledge, this is the first study investigating parents' healthcare use and its connection with their child's dental care use and quantifying the influence of parents' combined use of medical and dental care on their child's dental visit and preventive oral health service use. However, some limitations should be acknowledged. As this is a cross-sectional study, we do not have information on the timing of parent's and children's healthcare visits and cannot ascertain temporality or causality. Although MEPS is the best national data available to study healthcare utilization and has a robust panel design, the dental services used for children are reported by adult respondents and may suffer from reporting errors or bias. Although we controlled for several covariates, there may be some unmeasured confounders such as cultural beliefs or trust that could influence the results. Future studies should examine ways to improve dental care use among adults, identify reasons for differences in receiving preventive care services among children, and investigate ways to improve the delivery of these services in all age groups.

## Conclusions

This study provides estimates of dental utilization among children and its relationship with parents' healthcare use using robust MEPS data. Children's oral health utilization is strongly linked with parent's healthcare use and has a dose-response relationship. Although 2 out of 5 reported receipt of preventive services, only 1 in 5 children received evidence-based preventive services, including sealants or fluoride varnish, and there was a 3–5-fold difference in these dental care use outcomes based on parents' healthcare use. Oral health utilization among children remains low, especially for evidence-based preventive services. The information from this study suggests that improving healthcare use among parents can have important implications for children's dental care use.

## Data Availability

Publicly available datasets were analyzed in this study. This data can be found here: https://meps.ahrq.gov/mepsweb/data_stats/download_data_files.
