# Supplementary material for: Understanding the relationship between children's oral health utilization and parent's use of healthcare services
Source: Front Oral Health. 2025 May 13;6:1541045. doi: 10.3389/froh.2025.1541045 (PMC12106425; doi:10.3389/froh.2025.1541045)
Supplement: Supplementary file 1 [file Table1.docx]

Supplement Table: Adjusted logistic regression model examining the association between parent's healthcare utilization and Fluoride/Sealant receipt among children from 6-17 years, Medical Expenditure Panel Survey, 2017-2019

| **Characteristics** | **Fluoride/Sealant receipt** |
| --- | --- |
|  | **OR (95% CI)** |
| **Parent healthcare utilization** |  |
| Medical and Dental | **4.18 (3.15, 5.53)***** |
| Dental and No Medical | **2.81 (1.92, 4.12)***** |
| Medical and No Dental | **1.82 (1.38, 2.41)***** |
| No Medical and Dental visit | Ref |
| **Age groups** |  |
| 6-11 years | Ref |
| 12-17 years | **0.71 (0.59, 0.85)**** |
| **Gender** |  |
| Male | Ref |
| Female | 1.03 (0.87, 1.22) |
| **Race/ethnicity** |  |
| Non-Hispanic White | Ref |
| Hispanic | **0.66 (0.53, 0.82)**** |
| Non-Hispanic Black Only | **0.58 (0.44, 0.76)**** |
| Non-Hispanic Asian Only | **0.49 (0.33, 0.72)**** |
| Other or multiple Race | 1.19 (0.87, 1.63) |
| **Dental insurance** |  |
| Private dental | Ref |
| Public | 1.08 (0.84, 1.39) |
| Private health but no dental | 0.97 (0.76, 1.25) |
| Uninsured | 0.60 (0.33, 1.10) |
| **Health status** |  |
| Fair/poor | 1.16 (0.60, 2.25) |
| Good | 1.14 (0.91, 1.43) |
| Very good/excellent | Ref |
| **Region** |  |
| Northeast | 0.97 (0.75, 1.26) |
| Midwest | 1.27 (1.01, 1.61) |
| South | Ref |
| West | 1.22 (0.91, 1.63) |
| **Parent education** |  |
| <High School | **0.60 (0.39, 0.92)*** |
| High school | **0.61 (0.46, 0.81)**** |
| Bachelor's degree | 0.88 (0.68, 1.12) |
| Postgraduate degree |  |
| **Family poverty status** |  |
| Poor | **0.64 (0.45, 0.91)*** |
| Near poor | 0.95 (0.71, 1.27) |
| Middle income | 0.84 (0.69, 1.02) |
| High Income | Ref |
| **Parent employment** |  |
| Unemployed | 1.04 (0.84, 1.29) |
| Self-employed | 0.91 (0.69, 1.21) |
| Employed |  |
| **Number of children under 18 years in the family** |  |
| One | Ref |
| Two or three | **1.28 (1.06, 1.54)*** |
| Four or more | 1.23 (0.77, 1.96) |

Statistically significant p values are indicated as * p<0.05, **p<0.01, ***p<0.001
